# Antimalarial Evaluation of the Chemical Constituents of Hairy Root Culture of *Bixa orellana* L*.*

**DOI:** 10.3390/molecules19010756

**Published:** 2014-01-08

**Authors:** Bo Zhai, Julie Clark, Taotao Ling, Michele Connelly, Fabricio Medina-Bolivar, Fatima Rivas

**Affiliations:** 1 Arkansas Biosciences Institute, Arkansas State University, P.O. Box 639, State University, AR 72467, USA; 2 Department of Biological Sciences, Arkansas State, P. O. Box. 599, State University, AR 72467, USA; 3 Department of Chemical Biology and Therapeutics, St. Jude Children’s Research Hospital, Memphis, TN 38105, USA

**Keywords:** malaria, *Bixa orellana* L., stigmasterol, hairy root

## Abstract

Over 216 million malaria cases are reported annually worldwide and about a third of these cases, primarily children under the age of five years old, will not survive the infection. Despite this significant world health impact, only a limited number of therapeutic agents are currently available. The lack of scaffold diversity poses a threat in the event that multi-drug–resistant strains emerge. Terrestrial natural products have provided a major source of chemical diversity for starting materials in many FDA approved drugs over the past century. *Bixa orellana* L. is a popular plant used in South America for the treatment of malaria. In search of new potential therapeutic agents, the chemical constituents of a selected hairy root culture line of *Bixa orellana* L. were characterized utilizing NMR and mass spectrometry methods, followed by its biological evaluation against malaria strains 3D7 and K1. The crude extract and its isolated compounds demonstrated EC_50_ values in the micromolar range. Herein, we report our findings on the chemical constituents of *Bixa orellana* L. from hairy roots responsible for the observed antimalarial activity.

## 1. Introduction

Malaria remains a serious worldwide health problem, killing over half a million people per year, primarily children. The World Health Organization (WHO) has made considerable efforts to eradicate this infectious disease, but it is a constant effort in some parts of the world where vector control is not financially feasible. Nonetheless, in the past decade a 50% reduction of reported cases in 43 out of the 99 countries with ongoing transmission has been reported [1]. Both *Plasmodium vivax* and *Plasmodium falciparum* are found in Latin America, particularly in the Peruvian Amazon. A recent Peruvian *P. falciparum* genomic report identified selected regions of the genome containing genes underlying drug resistance and confirmed the parasites to be resistant to clindamycin [2]. The rise of resistance to other drugs is also an eminent threat. A drug cocktail regimen is recommended for the treatment of malaria to prevent the development of resistance, particularly to artemisinin, one of the most powerful components of this protocol. However, a growing number of countries in Southeast Asia have already reported artemisinin-resistant parasites [3], thus urging the scientific community to search for new antimalarial agents, especially new molecular scaffolds that may have novel mechanisms of actions.

In line with the fact that natural products continue to play a critical role in drug discovery [4] and as part of our continuing research efforts to study the medicinal properties of South American terrestrial plants, we launched an investigation on the constituents of *Bixa orellana* L.* (B. orellana)* hairy root culture. South America has rich plant biodiversity and Native Americans have a long standing tradition of utilizing natural product infusions to treat various ailments. *B. orellana*, a member of the Bixaceae family of flowering plants, commonly known as “annatto” in North America and “achiote” in Latin America, is widely cultivated for its condiment and medicinal properties. *B. orellana*‘s seeds are the main food colorant supply of bixin and norbixin [5] as well as a broad range of volatile oils elucidated through mass spectroscopy, including (*Z*,*E*)-farnesyl acetate, occidentalol acetate, and spathulenol [6,7]. *B. orellana* has been reported to treat many diseases, including infectious diseases, diabetes, stomach ulcers, [8] and malaria [9,10]. These ethnopharmacology reports prompted us to investigate the compounds responsible for such antimalarial properties.

Progress in biotechnology techniques has allowed the development of a deep understanding of terpenoid biosynthetic pathways in plants and they are attractive platforms for biosynthesis of secondary metabolites. Secondary metabolite extraction methods, particularly from plants, are well-established industrially and would therefore require limited optimization to move a scaffold forward for lead optimization. Furthermore, the addition of growth media stressors has been successful utilized [11] generating larger quantities of a specific secondary metabolite or providing new privileged molecular entities independent of climatic and geographic parameters in a more flexible process, particularly in terms of scalability. Root-based cultures technologies such as hairy roots (which are transformed roots established through *Agrobacterium*-mediated transformation) have become popular sustainable production platforms generating specific secondary metabolites [11,12]. Hairy roots technologies advantages include genetic and biochemical stability, ability to produce the metabolites found in the mother plants, and the opportunity to discover new chemical entities. Utilizing a similar approach, *B. orellana* hairy root cultures were established at the Medina-Bolivar laboratory [13] and racemic methyl jasmonate (MeJA) was selected as the elicitor. The jasmonates are known for their ability to modulate plant growth, stress response, and development in tissue cultures [14]. Jasmonic acid (JA) is a naturally occurring small volatile molecule produced enzymatically from linolenic acid in plants to serve as a signaling molecule that induces genes responsible for protein inhibition, seed and vegetative protein storage and metabolism, and secondary metabolite biosynthesis such as alkaloids in different plant species [15]. Although the jasmonates are ubiquitously present in various plant tissues, their presence in *B. orellana* has not been reported. Herein, we report the isolation and structural elucidation of the major extract constituents of *B. orellana* hairy roots after MeJA treatment and their antimalarial evaluation.

## 2. Results and Discussion

### 2.1. Structure Elucidation

**Figure 1:**
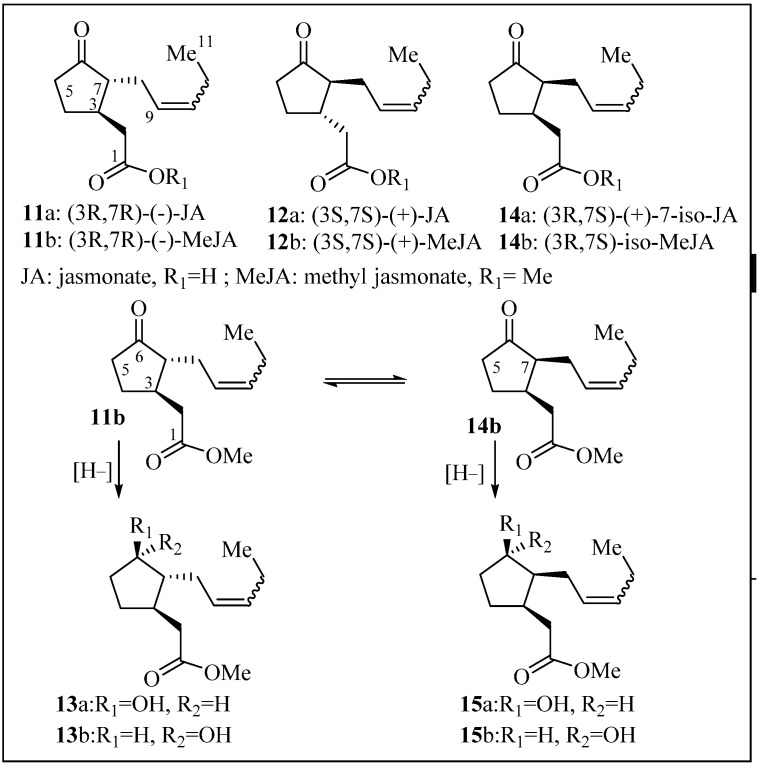
Naturally occurring jasmonate isomers and synthetic isomers.

**Figure 2:**
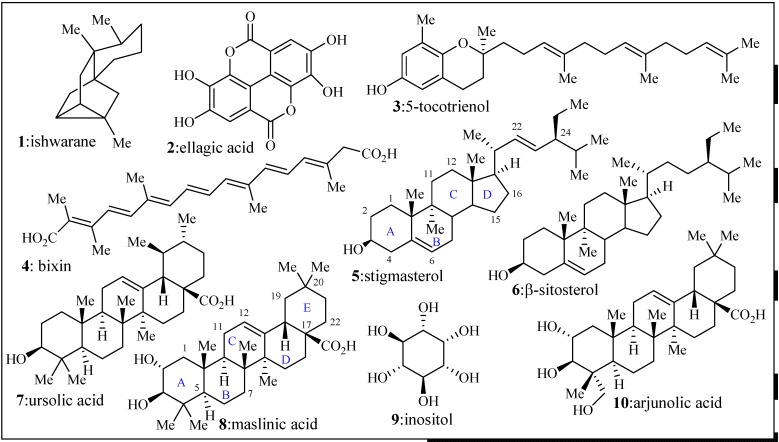
Isolated natural products from *B. orellana* hairy root extracts.

*Bixa orellana* hairy root line 15 [13] was cultured in a modified liquid Murashige and Skoog medium (MSV) for 15 days, and prior to harvest, the spent medium was removed and replaced with fresh MSV medium with MeJA (100 µM). Cultures were incubated for an additional 24 h. The jasmonates are structurally characterized by a cyclopentanone ring with two chiral centers (C-3 and C-7). The naturally occurring pair of enantiomers along with their diastereoisomers compounds **11–15** [(3*R*, 7*R*)-(−)-JA, (3*S*, 7*S*)-(+)-JA (JA), and (3*R*, 7*S*)-iso-7(+)-JA] have been reported in various plants and are shown in Figure 1 [16]. The protein targets of these natural compounds have been described as JIP-6 and JIP-23 (jasmonate–induced protein of 6 kDa and 23 kDa, respectively) in barley leaves, but their role remains unclear in other plants. Compound **11b** can potentially epimerize to **14b** and if ketone C6 is reduced, four diastereoisomers can be formed. It was anticipated that the culture medium could afford the free jasmonic acids, but it was unknown whether the epimerization at C7 would occur (**14b**, Figure 1). Under our experimental conditions, compound **14b** was not observed, nor its diastereoisomers (**15a**/**15b**). After MeJA treatment, the roots were filtered from the aqueous media and allowed to air-dry. The inorganic medium was extracted with EtOAc, and the solvent evaporated to afford crude yellow syrup containing ishwarane (Figure 2), methyl cucurbate (compound **13b**), jasmonic acid, MeJA, and volatile oils (not isolated, recorded by LCMS). The culture medium was neutral and no epimerization of **11b** to **14b** was detected, with or without the hairy roots. MeJA did not hydrolyze or decompose upon standing in the culture media (24 h). Traces of MeJA were recovered from the culture media, but it had mostly converted to compound **13b** and JA, in alignment with similar reports [17]. No traces of diastereoisomer **13a** were observed suggesting the conversion of **11b** to **13b** is an enzymatic reduction. Chemical reduction of MeJA with NaBH_4_ in MeOH afforded compounds **13b** and **13a** (3:2) quantitatively, which served to established stereochemistry.

After the root tissue was dried and minced, its ethanolic extracts provided several compounds that were characterized as known compounds **1–10** (ishwarane, ellagic acid, δ-tocotrienol, bixin, stigmasterol, β-sitosterol, inositol, ursolic acid, maslinic acid, and arjunolic acid respectively). A few amino acids (Trp, Phe, and Thr) were also present in this crude mixture, but they were not isolated individually. Compounds **5–10** were easily observed by crude NMR of the extract due to their high abundance (Figure 2).

**Figure 3:**
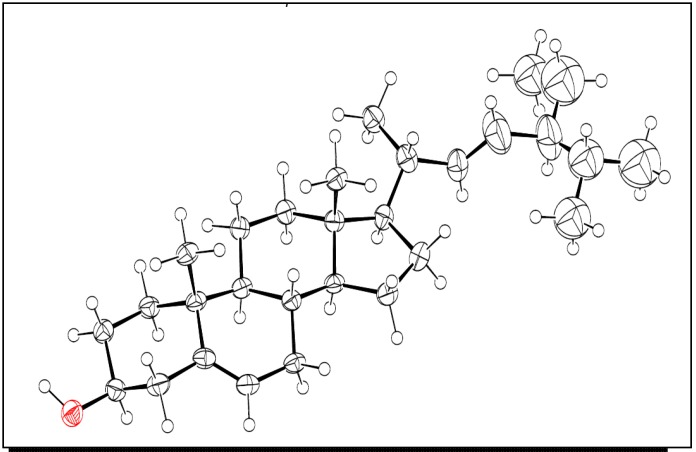
X-ray structure of compound **5**.

Compound **5** was present in large quantities and during isolation, it solidified to afford suitable crystals for X-ray analysis (Figure 3). This data set has been deposited and can be obtained at Cambridge Crystallographic Data Center (CCDC 892310). It was noted that only traces of bixin were detectable at this growth stage of the culture. Recent work of *B. orellana* roots cultured in the presence of light for 75 days produced annatto pigment in high quantities [18].

The sterols are attractive molecules due to their natural sources, and therefore potential for chemical derivatization to improve selectivity and potency. Recent studies have shown that tormentic acid (same core as maslinic acid, but bearing a methyl and a hydroxyl group at C-19, Figure 2) and β-sitosterol (compound **6**) have been tested in both *in vivo* and *in vitro* as antimalarial agents and these compounds were extracted from *Cecropia pachystachya* root from plant materials isolated during different years [19]. The *in vivo* data, mice infected with W2 strain of *P. falciparum* showed that both tormentic acid and β-sitosterol were active at 15 mg/Kg [19].

Our efforts highlight that sterols can directly be isolated from the hairy roots of *B. orellana* as a natural source, which can be mass produced for further derivatization/structure activity relationship studies and ultimately target identification.

### 2.2. Anti-Malarial Activity

The crude extract from culture medium and roots (compound **17**), a combination of MeJA derivatives (compound **16**), and its individual isolated components were tested *in vitro* for anti-plasmodium activity against malaria strains 3D7 and K1, as described in previously developed assays [20]. Pure compounds were prepared as 10 mM or 2 mM stock solutions in DMSO.

**Figure 4:**
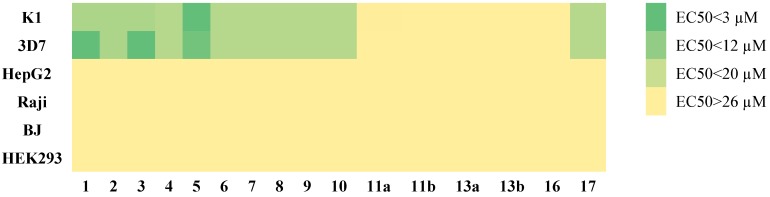
Anti-malarial heat map of compounds **1–17** and cytotoxicity panel.

The crude extract of *B. orellana* hairy roots (compound **17**) and its individual components displayed antimalarial properties in the 15–20 µM range and no cytotoxicity was observed at the measured concentrations in the mammalian cell lines utilized for this experiment (EC_50_ > 26 µM) as shown in Figure 4. MeJa derivatives did not show significant activity against the parasites in their pure state or as a mixture (compound **16**) to evaluate synergism. The crude extract (compound **17**) was tested based on mass (5 mg of the dried crude extract dissolved in 1 mL of DMSO) in a serial dilution fashion, and it was active only at the highest concentration with no effect on the mammalian cell panel. It was plotted against the other constituents in the heat map for comparison purposes. Compound **1**, **3** and **5** showed promising antimalarial activity, and compound **5** showed the highest potency on both K1 and 3D7 strains with EC_50_ of 5 µM and 3 µM respectively. Derivatization of these compounds will provide further understanding on their potential mode of action.

## 3. Experimental

### 3.1. Reagents and Instruments

Reagents were purchased at the highest commercial quality and used without further puriﬁcation unless otherwise stated. Reactions were monitored by thin-layer chromatography (TLC) carried out on 0.25 mm E. Merck silica gel plates (60F-254, Merck Millipore, Darmstadt, Germany) using UV light as visualizing agent, an ethanolic solution of anisaldehyde and heat as developing agents. Reactions were also monitored using Agilent 1100 series LC-MS (Agilent Technologies, Santa Clara, CA, USA) with UV detection at 254 nm and a low resonance electrospray model (ESI). Purification of title compounds was accomplished by flash column chromatography using E. Merck silica gel (60, particle size 0.040–0.063 mm) or Biotage Isolera Four (Biotage, Charlotte, NC, USA) with normal phase silica gel. ^1^H and ^13^C-NMR spectra were recorded on Bruker AV-400 or DRX-500 MHz NMR spectrometer instruments (Bruker, Billerica, MA, USA) and calibrated using residual undeuterated solvent (CDCl_3_: δ_H_ = 7.26 ppm, δ_D_ = 77.16 ppm; CD_3_OD: δ_H_ = 3.31 ppm, δ_C_ = 49.00 ppm) as an internal reference. The following abbreviations were used to designate the multiplicities: s = singlet, d = doublet, m = multiplet, br = broad. Infrared (IR) spectra were recorded on a Perkin-Elmer100 FT-IR spectrometer (Perkin-Elmer Life and analytical Sciences, Waltham, MD, USA). High resolution mass spectra (HRMS) were recorded on an Agilent ESI-TOF (time of ﬂight) mass spectrometer using MALDI (matrix-assisted laser desorption ionization) or ESI (electrospray ionization) or a Waters Xevo G2 Q-ToF mass spectrometer (Waters, Milford, MA, USA). Compounds were analyzed using electrospray ionization in positive-ion mode. Purity of final compounds was >95% based on analytical HPLC and NMR analysis. Yields refer to chromatographically and spectroscopically (^1^H-NMR) homogeneous materials.

### 3.2. Plant Material

Previously established *B. orellana* hairy root line 15 [13] was kept on solid MSV medium [21] before subculture and scale-up. The hairy root culture was maintained in liquid MSV medium with gentle shaking (orbital shaker, 90 rpm) at 28 °C in the dark for 15 days. On day 15, the spent medium was replaced with fresh MSC medium with MeJA (final concentration 100 µM) for 24 h. Hairy root materials were separated from the media and allowed to air-dry before extraction.

### 3.3. Extraction and Isolation

The *B. orellana* roots (10.6 g fresh weight) were filtered from the aqueous media, and allowed to air-dry before mincing and extracting them with a Soxhlet apparatus in refluxing isopropanol for 24 h. The inorganic media was extracted with EtOAc (250 mL × 3) and the solvent evaporated to afford crude yellow syrup, which was purified separately from the root extract. The major isolated components from this syrup were ishwarane, methyl jasmonate, methyl cucurbate, jasmonic acid, and inositol. The roots provided the following major compounds: iswarane, ellagic acid, δ-tocotrienol, bixin, stigmasterol, β-sitosterol, inositol, ursolic acid, maslinic acid, and arjunolic acid. All compounds were identified by 1D and 2D NMR and mass spectroscopy and TLC samples compared with commercially available standards.

### 3.4. Spectral Data for Compound **1**, **5**, **13a** and **13b**

*(2aS,6S,6aR)-1,6,6a-trimethyldecahydro-1,2a-methanocyclopropa[b]naphthalene* (**1**). Colorless oil, recently re-isolated showed identical NMR spectra [22]. ^1^H-NMR (400 MHz, CDCl_3_) δ 2.07 (m, 2H), 1.66–1.62 (m, 3H), 1.51 (bs, 1H), 1.48–1.45 (m, 2H), 1.31–1.33 (m, 1H), 1.14 (m, 2H), 1.12 (s, 3H), 1.01–0.94 (m, 2H), 0.79 (m, 1H), 0.77 (s, 3H), 0.70 (d, *J* = 6.6 Hz, 3H); 0.45 (bs, 1H); ^13^C-NMR (101 MHz, CDCl_3_) δ 43.91, 39.2, 38.66, 35.83, 35.64, 34.63, 33.59, 30.84, 23.95, 22.99, 22.46, 20.31, 19.82, 16.73, 16.55. HRMS (ESI-TOF) calcd for C_15_H_24_ ([M + H]^+^): 205.1956, found: 205.1953.

*(3S,9S,10S,13R,17R)-17-((2R,5S,E)-5-ethyl-6-methylhept-3-en-2-yl)-9,10,13-trimethyl-2,3,4,7,8,9,10,11,12,13,14,15,16,17-tetradecahydro-1H-cyclopenta[a]phenanthren-3-ol* (**5**). Off white crystals with a light yellow tint. ^1^H-NMR (400 MHz, CDCl_3_) δ 2.08, 96 (d, J = 1.3 Hz, 1H), 7.46–7.41 (m, 2H), 7.36 (dd, J = 1.8, 0.9 Hz, 1H), 7.19–7.13 (m, 1H), 4.12 (qd, J = 7.1, 1.0 Hz, 2H), 4.03–3.98 (m, 2H), 2.33 (dd, J = 10.9, 4.0 Hz, 2H), 1.82 (d, J = 7.5 Hz, 2H), 1.70 (d, J = 7.9 Hz, 2H), 1.51 (s, 2H), 1.25 (td, J = 7.1, 1.2 Hz, 3H); ^13^C-NMR (101 MHz, CDCl_3_) δ 140.77, 138.33, 129.29, 121.71, 71.82, 56.90, 55.98, 51.27, 50.19, 42.34, 42.24, 40.52, 39.71, 37.29, 36.54, 31.93, 31.69, 28.95, 25.44, 24.40, 21.25, 21.11, 19.43, 19.02, 12.28, 12.08. HRMS (ESI-TOF) calcd for C_30_H_51_O ([M + H]^+^): 427.3940, found: 427.3936. [α]_D_: = −40.5 (20 °C, c = 1.0, EtOH). M.p.: 167.8–168 °C.

*Methyl 2-(3-hydroxy-2-(pent-2-en-1-yl)cyclopentyl)acetate* (**13**)

In a 50 mL round bottom flask, methyl jasmonate (50 mg, 0.22 mmol, 1 eq.) was dissolved in anhydrous MeOH (2 mL) along with NaBH_4_ (34 mg, 0.89 mmol, 4 eq.) at −10 °C. The reaction mixture was stirred under nitrogen atmosphere for 1 h and brought to room temperature. Reaction was monitored by TLC and when completed, it was quenched with NH_4_Cl_(aq),_ extracted with EtOAc (3 × 50 mL), and washed with brine (50 mL × 2). The organic phase was dried over MgSO_4_, filtered, and concentrated. Column chromatography afforded the two compounds described below in a 3:2 ratio (a: 28 mg and b: 20 mg, 0.21 mmol, 97%).

*Methyl 2-((1R,2R,3R)-3-hydroxy-2-(pent-2-en-1-yl)cyclopentyl)acetate* (**13a**). Colorless oil. ^1^H-NMR (400 MHz, CDCl_3_) δ 5.44 (dd, *J* = 11.2, 7.2 Hz, 2H), 3.90 (d, *J* = 5.2 Hz, 1H), 3.66 (s, 4H), 2.55 (dd, *J* = 15.3, 4.9 Hz, 1H), 2.29 (dd, *J* = 15.2, 8.8 Hz, 1H), 2.18–2.01 (m, 10H), 1.86 (dd, *J* = 10.2, 7.4 Hz, 3H), 1.60 (s, 2H), 1.47 (s, 2H), 0.97 (t, *J* = 7.5 Hz, 4H). ^13^C-NMR (101 MHz, CDCl_3_) δ 173.78, 132.87, 127.44, 76.84, 74.39, 51.55, 51.08, 39.25, 38.77, 33.22, 29.44, 25.58, 20.68, 14.33. HRMS (ESI-TOF) calcd for C_13_H_23_O ([M + H]^+^): 227.1647, found: 227.1647. IR: 3390, 2900, 2820, 2316, 2297, 1704, 1411, 1347, 1272, 1239, 1178, 1145, 1048.

*Methyl 2-((1R,2R,3S)-3-hydroxy-2-(pent-2-en-1-yl)cyclopentyl)acetate* (**13b**). Colorless oil. ^1^H-NMR (400 MHz, CDCl_3_) δ 5.53–5.30 (m, 2H), 4.22 (s, 1H), 3.67 (s, 3H), 2.54 (d, *J* = 10.4 Hz, 1H), 2.30–2.00 (m, 7H), 1.95–1.81 (m, 1H), 1.63 (td, *J* = 5.9, 2.5 Hz, 1H), 1.51–1.18 (m, 4H), 0.97 (t, *J* = 7.5 Hz, 3H). ^13^C-NMR (101 MHz, CDCl_3_) δ 174.07, 133.98, 127.09, 78.88, 54.01, 52.00, 51.92, 40.45, 40.03, 33.68, 30.51, 30.14, 29.49, 21.07, 14.66. HRMS (ESI-TOF) calcd for C_13_H_23_O ([M + H]^+^): 227.1647, found: 227.1640. IR: 3359, 2900, 2877, 2821, 1703, 1411, 1345, 1237, 1180, 1148, 1062.

### 3.5. X-ray Crystallography of Compound **5**

Suitable colorless crystals were obtained upon column chromatography purification. X-ray data was deposited with the Cambridge Crystallographic Data Center (CCDC) and it is available under access number CCDC 892310. 

### 3.6. Antimalarial Test

All assays were performed in 384 well format and used less than 250 nL of test compound per well. To measure cell viability, a CellTiter-Blue Viability Assay (Promega, Madison, WI, USA) was used to determine the metabolic capacity of cells by their ability to reduce the indicator dye resazurin to resorufin. 50 μL of CellTiter-Blue Reagent diluted 5-fold in FBS-containing medium was added to compound-challenged cells after removal of medium. Cells were incubated with the reagent for 1 h at 37 °C in a 5% CO_2_ atmosphere, and fluorescence was recorded (560_Ex_/590_Em_).

#### Plasmodium falciparum

Parasites were grown in Petri dishes in the presence of fresh group O-positive erythrocytes (hematocrit 4%–6%) in RPMI 1640 (pH 7.3) supplemented with 0.5% AlbuMAX II, 25 mM HEPES, 25 mM NaHCO_3_, 100 μg/mL hypoxanthine, and 5 μg/mL gentamycin. Cultures were incubated at 37 °C with 5% O_2_ and 5% CO_2_. Compounds were prepared as 10 mM stock solutions in DMSO (except compound 18) to measure EC_50_. The stock solutions were plated in 384-well plates, serially diluted (1:3) in DMSO. Compounds were then transferred to a 384-well assay plate containing 20 μL of RPMI 1640 with 5 μg/mL gentamycin by using a 384-head pin tool. Twenty μL of a synchronized culture suspension (1% rings, 4% hematocrit) was added to each well (final hematocrit and parasitemia, of 2% and 1%, respectively). The final DMSO concentration was 0.65%. Assay plates were incubated for 72 h and parasitemia was measured by using a previously described DNA stain-based assay [20]. The MAR3D7 growth inhibition assay was used for the chloroquine-sensitive 3D7 strain of *P. falciparum*, and quantified growth by using DNA dyes. The experiment required a total of 96 h, including a 72 h incubation with test compounds. Positive controls were chloroquine and mefloquine. The MARK1 growth inhibition assay was used for the K1 strain of *P. falciparum*, known to be resistant to both chloroquine and pyrimethamine due to expression of the chloroquine resistance transporter and upregulation of the multi-drug resistance efflux pump. Growth was quantitated by using DNA dyes. The experiment required a total of 96 h, including a 72 h incubation with test compounds. Positive controls were chloroquine and mefloquine.

### 3.7. Cytotoxicity Assays

The human cell lines used in cytotoxicity assays were HepG2, BJ, RAJI, and HEK293 (purchased from the American Type Culture Collection, ATCC, Manassas, VA, USA) and were cultured according to ATCC recommendations. The HEPG2 human liver carcinoma cell line is a widely accepted model for studies of cytotoxicity. BJ is a normal human foreskin fibroblast stable cell line; HEK293 is a transformed human embryonic kidney cell line, also a well established for studies of general cytotoxicity. The RAJI Burkitt lymphoma cell line is the most sensitive of the four cell lines because of its rapid doubling time. Cells were grown in RPMI 1640 medium containing 10% FBS, 2 mM l-glutamine, and 1% penicillin/streptomycin (pen/strep) until nearly confluent.

Exponentially growing cells (RAJI, BJ, HepG2, and HEK293) respectively 1,200, 1,000, 400, 400 were plated per well (30 μL) in white polystyrene flat bottom sterile 384-well tissue culture treated plates (Corning, Tewksbury, MA, USA), and incubated overnight at 37 °C in a humidified 5% CO_2_ incubator. DMSO inhibitor stock solutions were pin-transferred (V&P Scientific, San Diego, CA, USA) the following day. Plates were placed back in the incubator for 72 h incubation and equilibrated at room temperature for 20 min before addition of 25 μL Cell Titer Glo (Promega) to each well. The positive controls were staurosporine (25 μM) and a toxic quinoline generated in-house. Plates were shaken on an orbital shaker for 2 min at 500 rpm. Luminescence was read after 15 min on an Envision plate reader (Perkin Elmer, Waltham, MA, USA).

## 4. Conclusions

This study of *B. orellana* hairy root demonstrates the power of natural product mining through hairy root culture technologies to access complex secondary metabolites as a valuable alternative to chemical synthesis. Utilizing this method, sufficient quantities of compounds **1–10** were obtained and these compounds were evaluated for their antimalarial properties. Compound **5** (stigmasterol) was identified as the most potent compound lead, the other chemical constituents of *B. orellana* hairy roots showed modest antimalarial properties in 3D7 and K1 strains with low toxicity in mammalian cells, thus provide a new potential source for further development.
